# Congenital Chloride Diarrhea Presenting With Polyhydramnios and Preterm Labor at 26 Weeks of Gestation: Prenatal and Neonatal Management

**DOI:** 10.7759/cureus.117296

**Published:** 2026-10-01

**Authors:** Fatima El Mangoub, Abdelhamid Benlghazi, Bouhtouri Yassine, Mly Mehdi Elhassani, Kouach Jaouad

**Affiliations:** 1 Department of Obstetrics and Gynaecology, Mohammed V Military University Hospital, Faculty of Medicine and Pharmacy, Mohammed V University, Rabat, MAR

**Keywords:** congenital chloride diarrhea, diarrhea, neonatal onset diarrhea, polyhydramnios, slc26a3

## Abstract

Congenital chloride diarrhea (CCD) is a rare autosomal recessive disorder linked to a dysfunction of the intestinal ion transporter. Its prenatal diagnosis relies on the combination of polyhydramnios and dilated fetal intestinal loops on ultrasound. We report the case of a 26-year-old woman, gravida 3, para 1, admitted at 26 weeks of gestation for threatened preterm labor due to acute polyhydramnios. Conservative management allowed the pregnancy to continue until 34 weeks of gestation, at which point a cesarean section was performed due to acute fetal distress. The newborn presented with watery diarrhea from birth. Initial intravenous rehydration with normal saline, combined with appropriate fluid and electrolyte supplementation, corrected the dehydration, electrolyte imbalance, and metabolic alkalosis, leading to a favorable clinical outcome.

## Introduction

Congenital chloride diarrhea (CCD) is a rare genetic enteropathy characterized by impaired active chloride absorption in the ileum and colon [1]. Prenatally, fetal excretion of hyperchloremic liquid stools leads to secondary polyhydramnios and progressive dilation of the fetal digestive tract. Early recognition of these ultrasound signs is essential to prevent major obstetric complications (particularly prematurity induced by uterine distension) and to anticipate neonatal fluid and electrolyte resuscitation at birth [2]. We present a case of CCD revealed at 26 weeks of gestation by polyhydramnios and threatened preterm labor.

## Case presentation

We report the case of a 26-year-old woman, gravida 3, para 1, with no reported consanguinity in the couple. Her gynecological and obstetric history included a first pregnancy terminated early by aspiration for a missed miscarriage, followed by a full-term cesarean delivery due to dystocia, delivering a healthy male infant weighing 3,000 g. The current pregnancy had progressed without any abnormalities in blood tests or blood pressure, and the oral glucose tolerance test was normal at 24 weeks of amenorrhea. Initial first-trimester screening at 10 weeks of amenorrhea demonstrated a normal nuchal translucency. Subsequent detailed anatomical scans at 17 and 24 weeks revealed no structural fetal anomalies or amniotic fluid abnormalities.

At 26 weeks of gestation, the patient presented to the emergency department with acute, excessive uterine distension and uterine contractions consistent with threatened preterm labor. Clinical examination revealed significant uterine enlargement; however, the patient remained symptom-free, reporting no dyspnea. Speculum examination was unremarkable, and digital vaginal examination showed a mid-length cervix. The ultrasound performed upon admission revealed marked polyhydramnios (amniotic fluid index or AFI = 26 cm), clinically correlated with the sudden increase in uterine height, as well as dilation of the fetal intestinal loops with anechoic contents, devoid of any signs of mechanical obstruction or associated peritoneal calcification. This ultrasound presentation, immediately suggestive of congenital chloride diarrhea, guided the entire management strategy (Figure 1).

**Figure 1:**
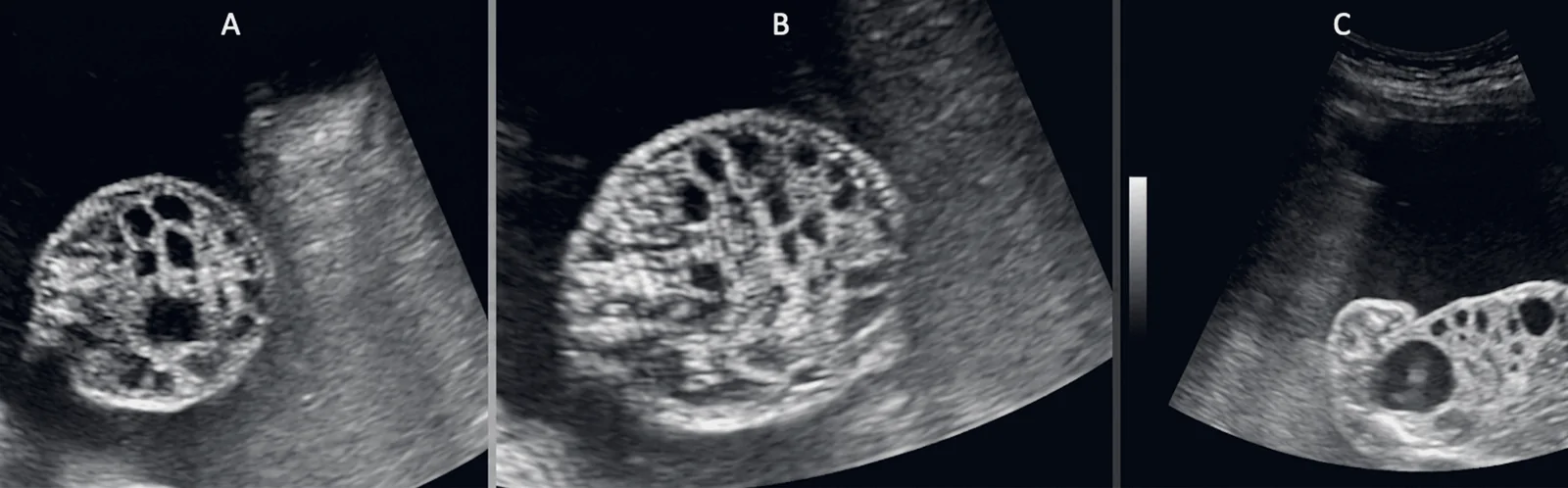
Ultrasound images at 26 weeks of gestation demonstrating multiple, fluid-filled, dilated bowel loops: (A) Axial section of the fetal abdomen showing numerous dilated bowel loops. (B) Axial section showing progressive continuity of the dilated segments. (C) Sagittal section displaying distal bowel dilation extending down to the rectum.

Certain complementary investigations commonly reported in the literature were intentionally not performed in this specific context. Amniocentesis, which theoretically allows for molecular analysis of the SLC26A3 gene, karyotyping or chromosomal microarray analysis, and amniotic chloride measurement, was ruled out due to the considerable iatrogenic risk it posed in a patient with threatened preterm labor and an already significantly distended uterus; the procedure carried a risk of premature rupture of membranes, potentially triggering irreversible labor. Furthermore, obtaining genetic or biochemical confirmation would not have altered the immediate obstetric management, which was based primarily on tocolysis and appropriate fetal lung maturation, and the timeframes inherent in molecular biology far exceeded the time frame for making an acute decision. Similarly, second-line fetal magnetic resonance imaging was abandoned, as high-resolution ultrasound data were sufficient to characterize bowel loops with anechoic fluid content in a manner sufficiently suggestive to rule out a complex mechanical obstruction or meconium peritonitis, whereas such an examination would have imposed a potentially harmful prolonged supine position.

Immediate treatment involved tocolysis with a calcium channel blocker (Nicardipine 2.4 mg/h) and fetal lung maturation corticosteroid therapy with betamethasone. Once the obstetric threat was stabilized, a close-monitoring protocol was implemented based on weekly ultrasound assessments of amniotic fluid volume, fetal intestinal caliber, and hemodynamic status via Doppler ultrasound of the umbilical and middle cerebral arteries. A joint prenatal consultation with the neonatology team informed the parents of the expected postnatal care pathway, including the need for immediate intravenous access and blood and stool tests, and reassured them of an overall favorable long-term prognosis, provided that appropriate electrolyte replacement is maintained.

This conservative strategy allowed the pregnancy to be prolonged for an additional eight weeks, with delivery ultimately occurring via a repeat cesarean section at 34 weeks of gestation due to acute fetal distress, resulting in the birth of a male infant weighing 3,000 g with Apgar scores of 10 at 1 and 5 minutes.

From the first hours of life, the newborn presented with profuse, watery diarrhea that persisted for six days, placing the infant at immediate risk of severe dehydration. Baseline laboratory investigations performed immediately after birth, prior to the initiation of intravenous therapy, confirmed marked metabolic alkalosis with severe hypochloremia and hypokalemia (pH > 7.50, low serum chloride and potassium levels), alongside preserved renal function parameters. Stool electrolyte testing demonstrated high fecal chloride concentrations exceeding 90 mmol/L, definitively confirming the diagnosis of congenital chloride diarrhea (CCD).

Enteral feeds were initially withheld during this acute phase of profuse diarrhea to allow for prompt hemodynamic and metabolic stabilization. Intravenous fluid and electrolyte replacement with chloride-enriched saline was initiated immediately, leading to progressive correction of gastrointestinal losses, cessation of diarrhea by the sixth day of life, and stabilization of the infant's clinical status. Once normochloremia, normokalemia, and adequate hydration were restored, oral enteral feeding (breast milk or standard infant formula) was gradually introduced alongside targeted oral sodium and potassium chloride supplementation. Enteral feeds were well-tolerated, allowing for the progressive weaning of intravenous fluids while maintaining steady infant weight gain and metabolic stability.

## Discussion

CCD is a rare autosomal recessive disorder resulting from mutations in the SLC26A3 gene (formerly known as DRA, "down-regulated in adenoma"), which encodes a Cl⁻/HCO₃⁻ exchanger expressed in the intestinal epithelium, primarily ileal and colonic. This transport defect leads to massive and uncontrolled fecal chloride loss, causing profuse watery diarrhea in utero. This diarrhea accumulates in the fetal intestinal lumen and clinically manifests as severe polyhydramnios, dilation of the intestinal loops, and, as in our case, preterm labor [3].

CCD is particularly common in Finland, Poland, Saudi Arabia, and Kuwait. Populations with SLC26A3 mutations have been identified [4], but sporadic cases and various mutations have been reported in many other populations, including Iberian and Central European populations [5-8]. More than 50 mutations of the gene are currently listed, with no clear genotype-phenotype correlation, which explains the variability in severity observed from one patient to another [5].

Prenatal diagnosis of CCD remains difficult because polyhydramnios associated with dilated fetal intestinal loops initially suggests, on ultrasound, a high intestinal obstruction (duodenal or jejunoileal atresia), a meconium plug, or Hirschsprung's disease [9]. Several authors have thus reported cases initially misdiagnosed as intestinal atresia or neonatal obstruction, delaying specific management [10]. The ultrasound features suggestive of CCD are the absence of frank peristalsis typical of mechanical occlusion, the presence of multiple dilated loops with homogeneous liquid content without a clear hydro-aeric level, and above all the demonstration, in color Doppler, of a "back and forth" flow of intraluminal content corresponding to the passage of diarrheal stools into the colonic lumen, a sign now recognized as almost pathognomonic [11-15].

Fetal magnetic resonance imaging, in addition to ultrasound, provides better morphological characterization of the dilated digestive tract, particularly when polyhydramnios makes ultrasound analysis difficult at the end of pregnancy. Several teams have highlighted its value in distinguishing congenital diarrhea from other causes of fetal bowel obstruction by analysis of the intraluminal signal and the topography of the dilation [16].

Diagnostic confirmation classically relies, in the neonatal period, on demonstrating a fecal chloride concentration greater than 90 mmol/L associated with hypochloremic and hypokalemic metabolic alkalosis, a condition initially described by Gamble and Darrow as early as 1945 under the term "congenital alkalosis with diarrhea" before the identification of the molecular mechanism [17,18]. During the prenatal period, measuring amniotic fluid chloride levels has been suggested as a diagnostic tool when concentrations exceed expected gestational norms; however, its clinical utility remains restricted by limited test availability [19].

Managing acute polyhydramnios in this setting requires balancing two distinct risks: the severe complications of early preterm birth versus the metabolic consequences of CCD. While delivery at 26 weeks exposes the fetus to substantial mortality (20-30%) and severe neurological and respiratory morbidities, extending gestation to 34 weeks dramatically improves neonatal survival (>95%) and limits prematurity complications. Consequently, the primary postnatal challenge shifts to managing acute dehydration and severe electrolyte imbalances caused by massive chloride-rich fecal losses, which carry high mortality if recognition is delayed. Neonatal management hinges on early and lifelong oral supplementation with sodium and potassium chloride to replace digestive losses and prevent long-term complications such as growth failure, nephropathy accompanied by hyperuricemia and nephrocalcinosis, and male infertility [20-22]. Ideally established prenatally, early diagnosis enables immediate postnatal treatment and avoids diagnostic delays, which frequently occur when the disease is misattributed to neonatal bowel obstruction [7,12].

Long-term follow-up of patients receiving appropriate replacement therapy is generally favorable, with normal growth and psychomotor development, provided that strict adherence to treatment and regular nephrological monitoring are maintained, owing to the risk of late renal complications [22]. Genetic counseling is also essential given the autosomal recessive mode of transmission, particularly in at-risk populations or in cases of a family history, as illustrated by descriptions of affected siblings [6,9].

## Conclusions

Congenital chloride diarrhea (CCD) must be actively considered in cases of acute, severe polyhydramnios accompanied by dilated fetal bowel loops. Often misdiagnosed as a mechanical bowel obstruction, precise sonographic evaluation, supported by fetal MRI when indicated, plays a central role in prenatal suspicion. Early identification transforms clinical outcomes by allowing planned delivery in a specialized tertiary center and immediate postnatal administration of sodium and potassium chloride supplementation, thereby preventing severe dehydration and renal impairment. Beyond immediate neonatal resuscitation, molecular confirmation via SLC26A3 gene sequencing remains essential for definitive diagnosis and autosomal recessive genetic counseling. Future perspectives should focus on integrating rapid non-invasive prenatal testing (NIPT) for single-gene disorders, standardizing post-discharge electrolyte monitoring, and establishing long-term pediatric cohorts to better define growth trajectories and renal outcomes in CCD patients.
